# A group-randomized controlled trial for health promotion in Girl Scouts: Healthier Troops in a SNAP (Scouting Nutrition & Activity Program)

**DOI:** 10.1186/1471-2458-10-81

**Published:** 2010-02-19

**Authors:** Richard R Rosenkranz, Timothy K Behrens, David A Dzewaltowski

**Affiliations:** 1 Department of Human Nutrition, Kansas State University, Manhattan, KS, USA; 2 Youth Health Behavior Research Laboratory, Kansas State University, Manhattan, KS, USA; 3 Department of Health Sciences, Univ. of Colorado, Colorado Springs, CO, USA; 4 Department of Kinesiology, Kansas State University, Manhattan, KS, USA

## Abstract

**Background:**

Girl Scouting may offer a viable channel for health promotion and obesity prevention programs. This study evaluated the effectiveness of an intervention program delivered through Girl Scout Junior troops that was designed to foster healthful troop meeting environments and increase obesity prevention behaviors at home.

**Methods:**

Seven Girl Scout troops were randomized to intervention (n = 3, with 34 girls) or standard-care control (n = 4, with 42 girls) conditions. Girls ranged in age from 9 to 13 years (mean 10.5 years). Intervention troop leaders were trained to implement policies promoting physical activity (PA) and healthful eating opportunities at troop meetings, and to implement a curriculum promoting obesity-prevention behaviors at home. The primary outcome variable was child body mass index (BMI) z-score. Secondary outcomes included accelerometer-assessed PA levels in troop meetings, direct observations of snack offerings, time spent in physically active meeting content, and leader encouragement of PA and healthful eating.

**Results:**

The intervention was delivered with good fidelity, and intervention troops provided greater opportunities for healthful eating and PA (x^2 ^= 210.8, p < .001), relative to control troops. In troop meetings, intervention troop leaders promoted PA (x^2 ^= 23.46, p < .001) and healthful eating (x^2 ^= 18.14, p < .001) more frequently, and discouraged healthful eating and PA less frequently (x^2 ^= 9.63, p = .002) compared to control troop leaders. Most effects of the intervention on individual-level variables of girls and parents were not significantly different from the control condition, including the primary outcome of child BMI z-score (F_1, 5 _= 0.42, p = .544), parent BMI (F_1, 5 _= 1.58, p = .264), and related behavioral variables. The notable exception was for objectively assessed troop PA, wherein girls in intervention troops accumulated significantly less sedentary (x^2 ^= 6.3, p = .011), significantly more moderate (x^2 ^= 8.2, p = .004), and more moderate-to-vigorous physical activity, (x^2 ^= 18.4, p < .001), than girls in control troops.

**Conclusions:**

Implementing a health promotion curriculum and supporting policies to provide more healthful environments in Girl Scout troop meetings appears feasible on a broader scale. Additional work is needed to bridge health promotion from such settings to other environments if lasting individual-level behavior change and obesity prevention remain targeted outcomes. Trial registration number: NCT00949637

## Background

In the United States, there has been roughly a three-fold increase in childhood obesity prevalence over the past forty years [1]. According to the most recent data from the National Health and Nutrition Examination Survey, 33.6% of children and adolescents aged 2 to 19 are now overweight or obese (at or above the 85^th ^percentile of relative weight for their age and gender), and 17.1% are obese (at or above the 95^th ^percentile) [2]. Obesity is associated with numerous negative health outcomes, and particular public health concerns have arisen with regard to children for insulin resistance syndrome, hypertension, dyslipidemia, chronic inflammation, increased blood clotting tendency, endothelial dysfunction, and hyperinsulinemia leading toward type 2 diabetes [3]. The following background sections offer a brief review of behavioral influences on pediatric obesity and settings for intervention that serves to inform the present study's health promotion approach.

### Influences on overweight and obesity in children

The American Academy of Pediatrics (http://www.aap.org, accessed July 10, 2009) has made several recommendations for children's obesity prevention and health improvement, and among these are: 1) Eat five fruits and vegetables per day; 2) Limit consumption of sugar-sweetened beverages; 3) Limit screen time to less than two hours a day; 4) Get one hour of physical activity a day; and 5) Regularly eat family meals together.

#### Fruit and vegetable consumption

The consumption of fruits and vegetables (FV) is negatively related to obesity, and researchers and practitioners often focus on boosting the intake of FV in both clinical obesity treatments and primary prevention efforts [4]. Among the numerous positive attributes of FV, is the fact that they contain abundant water and fiber, which may promote satiety and reduce overeating [5]. Studies have shown that enhancing availability and accessibility of FV increases consumption in children [6]. In school-based settings, both Sahota and colleagues and Müller and colleagues were able to modify FV consumption patterns, and the latter study achieved a corresponding indicator of obesity prevention [7,8]. Interventions designed to boost FV consumption in children have also been effectively delivered through scouting programs in earlier studies [9-11].

#### Sugar-sweetened beverage consumption

Many studies have identified the consumption of sugar-sweetened beverages (SSB) to be a risk factor for obesity [12,13]. James and colleagues used a randomized controlled trial to demonstrate the effectiveness of an intervention designed to decrease the consumption of SSB in primary school children [13,14]. The children received an educational program and music designed to teach them SSB-related oral health risks, and to limit their consumption of such drinks, replacing SSB with water. James' intervention showed short-term success in decreasing SSB consumption and the percentage of overweight and obese children. Recent pilot work by Ebbeling and colleagues has demonstrated effectiveness in replacing SSB with low-calorie beverages for obesity prevention in adolescents [15].

#### Television Viewing

Television (TV- one form of screen time) may promote childhood obesity through three avenues of influence: promotion of sedentary behavior, food advertising, and eating while watching TV [16]. Gortmaker and co-workers based much of their Planet Health intervention curriculum on the reduction of TV for 6th and 8th graders [17]. Planet Health achieved success in reducing TV viewing and the frequency of eating with TV. The intervention was effective for obesity prevention only for girls, and the effect was mediated by decreased TV viewing. In another study aiming to decrease TV viewing in third and fourth graders, Robinson enlisted the aid of parents and an electronic device to restrict the amount of TV time [18]. This study achieved success in changing TV behavior and preventing obesity for both boys and girls. Thus, interventions designed to limit children's TV viewing, and to eliminate the connection between eating and TV may be effective in preventing obesity.

#### Physical Activity

Increasing physical activity (PA) of children may be useful in public health interventions to prevent obesity [19-21]. Previous research studies have used scouting programs to boost PA in children [22,23]. Outcomes have been modest for these interventions, and many have successfully increased PA without a concomitant change in BMI or prevalence of obesity [24,25]. To prevent obesity and to gain other health benefits, regular opportunities for enjoyable PA is a desirable and useful component of health promotion efforts.

#### Family Meals

As societal eating patterns have shifted, children and parents of the 21st century may not be eating family meals (FM) together at the dinner table as in previous generations [26]. Instead, individuals are likely eating alone or in more casual fashion, mindlessly snacking while watching television [27]. Eating alone and with the TV may be associated with higher speed of eating and greater caloric consumption [28]. Although FM appears to be an important modifiable determinant of nutritional intake and children's weight status, few interventions have attempted to increase the frequency, or improve the quality of FM [29-31]. Potential barriers to bolstering FM include lack of time and skills needed to prepare food [32].

### Settings to Influence Health Behaviors for Obesity Prevention

According to a meta-analytic review, many obesity prevention interventions have not shown strong effectiveness [33]. Systematic reviews have been generally unsupportive of obesity prevention intervention effectiveness [34]. Interventions with larger effect sizes were brief, focused on weight control outcomes, and targeted younger children and adolescents. Some authors have argued for the necessity of including parents, but this approach has been difficult to achieve, and has led to limited success [35,8]. Many school-based interventions have failed to engage parents, or to achieve beneficial outcomes with this approach [24,36].

Obesity prevention interventions are frequently delivered in school settings, including a recent focus on after-school programs [37]. Some interventions have been implemented in community centers, churches, and youth clubs. Researchers have long recognized the importance of parents and the home environment on obesity prevention efforts, but the ability to bridge from institutional settings such as schools to parents and the home environment has been elusive for obesity prevention.

#### Girl Scouts

The Girl Scouts of the USA is a not-for-profit national organization that is a member of the world association of Girl Guides and Girl Scouts, and is dedicated to building the courage, confidence, and character of girls, to make the world a better place (http://www.girlscouts.org, accessed July 10, 2009). Girl Scouts may offer a viable channel for health promotion and obesity prevention interventions due to several inherent factors. First, the national organization is committed to promoting the health and well-being of girls, and several merit badges exist that reward girls for their efforts to improve knowledge and behavior related to PA, nutrition, and healthy living. Second, there is diversity amongst the members of Girl Scouts with regard to socio-economic status, race, and ethnicity. Third, the organization and troop leaders are focused on promoting youth development, and a system of socialization exists wherein the girls are expected to learn new skills, are empowered to make changes in their lives, and are asked to complete projects designed to demonstrate what they have learned. Several researchers have used Girl Scout troops to deliver interventions designed to promote health behaviors [11,22,23].

#### Rationale for the present study

Although a broad array of obesity prevention interventions currently exists, there are no published reports of randomized controlled trials targeting the promotion of healthful scout meeting environments or family meals. There are an assortment of multi-level interventions based in schools and other institutions attempting to bridge health promotion and obesity prevention effects to the home environment, but few of these interventions have explicitly attempted to impact the home environment by enhancing the skills of children within the institutional environment setting. Therefore, the purpose of this study was to evaluate an intervention designed to prevent obesity by modifying Girl Scout troop meeting environments, and by empowering girls to improve the quantity and/or quality of family meals in their home environments.

## Methods

### Study design

This study is a group-randomized controlled trial using a nested cohort design, with troops being the unit of randomization [38]. Our sample size and *a priori *power calculation were based on our research team's analogous group-randomized trial taking place in after school programs and powered to detect a 0.5 unit BMI change between 4 intervention and 4 control sites with 20 girls per site. Our *post-hoc *power calculations showed that the present group-randomized trial had 90% power to detect a primary outcome BMI-z score difference of 0.31 (roughly half the distance between the 85^th ^and 95^th ^percentiles) between intervention and control means, and 75% power to detect the same effect in our subgroup of overweight girls. Related *post-hoc *calculations showed limited power to detect differences in individual outcomes of reported PA (23% power to detect a difference of 60 minutes per day, 1.5 days per week) and FV consumption (20% power to detect a difference of one serving per day).

**Figure 1:**
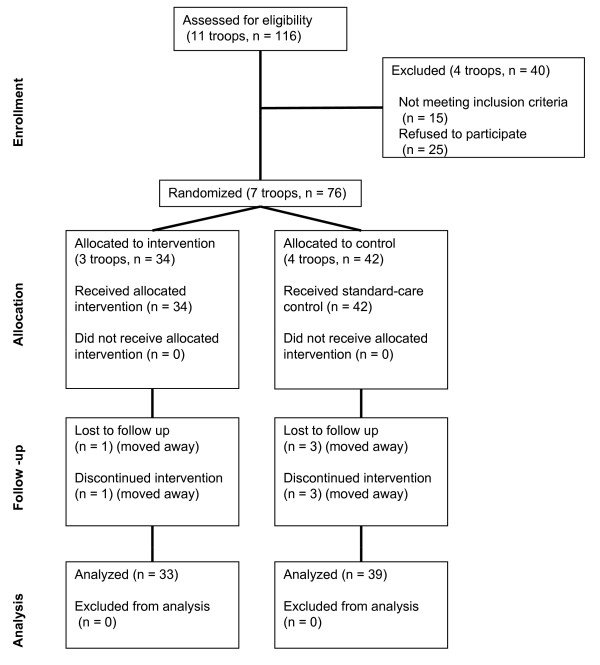
**CONSORT diagram showing the flow of participants through stages of randomized trial**.

Seven troops agreeing to participate completed a pretest time 1 assessment within a two-week period in October before randomization. Troops were stratified into large (n = 4) and small size troops (n = 3) and then according to a random number generator were randomized (by first author) within strata to the control or intervention conditions (see Figure 1). After being trained for implementation of the curriculum and supporting policies, the intervention troop leaders instituted the intervention components at the next scheduled troop meeting. A trained research assistant observed each troop during seven full meetings between time 1 (October, 2007) and time 2 (April, 2008) assessments to record troop meeting environmental variables, including leader health-promotion behaviors. At study commencement, research assistants were blind to condition of each troop. Following the seven observations, all troops underwent the time 2 assessment during a two-week period in April. The research protocol received approval from the IRB at Kansas State University (#4389).

### Sampling and Participants

#### Girl Scout Troops

To meet study inclusion criteria, the troop needed to be a registered Girl Scout Junior troop, consisting of girls in the 4^th ^and 5^th ^grades. To be registered, the troop leaders had to complete Girl Scout leader training and pass a criminal background check. Troops also needed to meet at least twice per month, have meeting facilities capable of allowing physical activity and food preparation. Troops also needed to have initial agreement of leaders and parents for the troop to participate in a research study. Exclusion criteria included troops not primarily composed of Girl Scout Juniors, not regularly meeting during the study period, or not having leader and parental consensus approval for troop participation. Participating troops earned $10 per girl, and individual families earned $10 for taking part in the study.

#### Girl Scouts and Parents

To meet study inclusion criteria at the individual level, girls had to be attending members of Girl Scouts in one of our included troops. All girls of participating troops were included for direct observation variables, and those with parental consent were included for the individual variables under study. Parents were included if they agreed to complete a questionnaire for each child attending one of the troops. Exclusion criteria included an inability to speak or read English (n = 1).

In the seven Girl Scout Junior troops, parental informed consents were obtained for all but one child (n = 76, 100% female). Of parents consenting for their child to participate (n = 72) a majority also participated by returning questionnaires (n = 68). Troops held meetings in one of three adjacent Midwestern towns, ranging in population from about 4,000 to 50,000. Troop meetings were held either weekly (2 troops) or bi-monthly (5 troops), and between one and two hours in duration. Meetings were held at the Girl Scouts organization's property (4 troops), at a troop leader's home (2 troops), or at a community center (1 troop). Troops ranged in size from 6 to 16 girls (mean = 11). Individual characteristics are presented in Table 1.

**Table 1:** Individual characteristics by troop assignment at Time 1

Demographic & Psychosocial Variables	Intervention Mean (SD)	Control Mean (SD)
Percent parents are college graduates	56.3	48.7
Percent lower socio-economic status	28.1	35.0
Percent Non-Hispanic Caucasian girls	79.4	75.0
Percent racial/ethnic minority girls	20.6	25.0
Authoritarian Parenting Scale Score^a^	31.1 (3.9)	32.9 (4.4)
Authoritative Parenting Scale Score^a^	38.8 (3.3)	38.6 (3.6)
Permissive Parenting Scale Score^a^	23.2 (3.9)	23.9 (4.1)
Family Cohesion Scale Score^b^	64.4 (10.4)	60.4 (12.2)
Children per household	2.7 (1.3)	2.4 (0.8)
Girl's Age	10.6 (1.1)	10.5 (1.3)

Note: ^a^Scale scores 10-50, higher numbers possessing more of the trait; ^b^Scale scores 16-80, higher numbers possessing more of the trait; no significant differences by condition

### Description of Intervention

Our intervention was based on core components of Social Cognitive Theory, including: Role modelling by peers, troop leaders, and parents; skill building through active mastery experiences; enhancement of self-efficacy and proxy efficacy through role playing and active mastery experiences; and reinforcement of behavior through verbal praise and merit badges [39]. The intervention consisted of three main components: 1) An interactive educational curriculum delivered by troop leaders; 2) Troop meeting policies implemented by troop leaders; and 3) Badge assignments completed at home by Girl Scouts with parental assistance. The educational curriculum consisted of eight modules, delivered over the course of about four months. This intervention curriculum is an expanded version of our previously published work used in summer programs [30].

Each module consisted of a discussion of intervention target behaviors, worksheet for goal setting and self-monitoring, physically active recreation session (e.g., walking, dancing, yoga, and active games), FV snack recipe preparation, FM role-playing, clean-up period, and description of the take-home assignment. The modules were designed to require 60-90 minutes to deliver, with flexibility allowed for specified program activities and module order. Troop leaders underwent two hours of training by the first author prior to intervention commencement. Regular and ongoing email and phone support took place throughout the intervention time period.

Target behaviors of the intervention included: 1) Frequent FM; 2) Parent-child shared PA; 3) Elimination of TV during mealtime; 4) Drinking water instead of SSB at mealtime; 5) Including FV in FM; 6) Practicing good manners during FM; 7) Helping parents prepare FM and cleaning up afterwards.

Troop meeting policies included: 1) Providing 15 minutes per meeting for physically active recreation; 2) Troop leaders participating in physically active recreation with girls; 3) Provision of a FV snack prepared by girls; 4) Troop leaders eating FV snack with girls; 5) Troop leaders verbally promoting PA, FV consumption in troop meetings and for home, and verbally promoting FM for home; and 6) Prohibition of SSB, candy, and TV watching during meetings.

### Process Measures

#### Evaluation Procedures

Over the course of the intervention period (October 2007 to April 2008) on seven occasions, a research assistant attended Girl Scout troop meetings. From the beginning to end of each meeting, the research assistant continuously directly observed aspects of the troop environment and activities, recording observations in a customized logbook.

Curriculum implementation was evaluated via observer reports (observation forms, described below) and troop leader self-evaluation forms. Troop leaders self-rated the degree of implementation for eight components of each troop meeting. Leaders responded on a three-point scale (zero to two), indicating no, partial, or full implementation for each curricular component.

#### Troop Environmental Observations

For each meeting, a trained research assistant recorded details of meeting context on a customized SNAP Session Form. This session form was patterned off SOPLAY, with observers noting the condition of the physical area for each session [40]. Session was defined as a period of time that the majority of girls were engaged in one activity. Transition to a new session began when 51% or more of the girls moved to a new activity. During snack, the research assistant completed a customized SNAP Snack Observation Form, noting all foods and drinks accessible to girls and troop leaders, including the number of girls and adults actually consuming each food product. If food or drink was accessible and consumed at periods outside the snack session, details were also noted on this snack form. Throughout the troop meeting, the research assistant used a customized SNAP Troop Observation Form to record the general structure, general content, knowledge content, and leader behavior relevant to promotion of PA and healthful eating. The research assistant used a portable timing device with vibrating alert to determine presence or absence of each condition and behavior every 60 seconds, for the duration of the troop meeting. The behavioral and environmental observation system and form were developed according to recommended guidelines for behavioral observation and patterned off SOFIT methodology [41,42]. Two research assistants were carefully trained to use all forms and observation techniques, and adequate inter-rater reliability (>90% agreement) was obtained prior to actual data collection.

#### Reliability estimates

Table 2 displays our study's reliability checks, where most variables achieved adequate reliability. The three PA promotion behaviors and three healthful eating promotion behaviors were later collapsed into "any physical activity promotion" and "any healthful eating promotion", which improved reliability substantially (percent agreement > 90).

**Table 2:** Inter-rater reliability statistics for troop environmental variables (based on 144 observed minutes)

	Percent Agreement	Intra-class correlation	Cohen's Kappa	Significance level
**Session Context**				
Free time or structured activity	100	1.000	1.000	P < .001
General meeting content	95.9	.977	.941	P < .001
PA educational content	88.8	.199	.099	P = .006
HE educational content	98.6	.920	.850	P < .001
				
**Troop Leader Behavior**				
PA verbal promotion	82.5	.083	.038	P = .438
PA physical promotion	97.2	.954	.911	P < .001
PA promotion out-of-troop	100	1.000	1.000	P < .001
Any PA promotion	98.6	.980	.960	P < .001
HE verbal promotion	94.4	-.026	-.012	P = .820
HE physical promotion	96.5	.000	**	**
HE promotion out-of-troop	97.2	.746	.588	P < .001
Any HE promotion	90.3	.542	.367	P < .001
No HE or PA promotion	89.5	.857	.746	P < .001

Note: PA = physical activity; HE = healthful eating; **Unable to compute values due to lack of variability in this observation

### Individual-Level Outcome Measures

#### Evaluation Procedures

For the time 1 (early October 2007) and time 2 (April 2008) assessments, a research assistant travelled to troop meetings where girls were assessed on height, weight, self-reported psychosocial influences, and health behaviors. Girls' height and weight assessments were carried out in semi-private settings without shoes or heavy clothing. Height was measured to the nearest millimeter, using a portable stadiometer (Seca Corp, Model #214 Road Rod, Hanover, MD). Weight was measured to the nearest 0.1 kg with high-precision electronic scales (Seca Corp, Model #770, Hanover, MD). For reliability, duplicate height and weight measurements were taken, and third measurements were taken if the first two differed by more than 5 mm or 0.5 kg. Girls completed identical questionnaires at times 1 and 2, administered according to a standardized script read by the first author. Parents completed a questionnaire outside of troop meeting times, before and after the intervention period.

#### Anthropometry

Body mass index (BMI) was calculated by dividing body weight (kg) by height (m) squared. BMI scores were converted to percentiles and z-scores (our primary outcome variable) using the age- and sex-specific LMS parameters from the CDC growth charts [43]. Participants were classified as overweight or obese, respectively, if their BMI equalled or exceeded the age- and sex-specific 85^th ^or 95^th ^percentile (z-scores of 1.036 or 1.645 respectively).

#### Accelerometry

Objective assessment of PA was obtained using the ActiGraph GT1M accelerometer (Shalimar, FL). At the beginning of the seven observed troop meetings (October through April), a research assistant placed an accelerometer on each girl's right hip, using an adjustable elastic belt. The assistant recorded the starting time and the identification number of the accelerometer worn by each girl. Scouts wore the accelerometer for the duration of their meeting attendance. Using a 30-second epoch, raw accelerometer counts were processed through a customized software program for determination of time spent in moderate-to-vigorous (MVPA; = 4 METs), vigorous (= 7 METs), moderate (4 - 6.99 METs), light (1.5 - 3.99 METs), and sedentary (< 1.5 METs) PA levels. The age-specific count thresholds corresponding to these intensity levels were derived from the MET prediction equation developed by Freedson and co-workers, and the appropriate count thresholds were divided by two to accommodate the 30-second epoch length [44]. Invalid wearing time during the meeting period was assessed by counting the number of consecutive zero counts accumulated in strings of 10 minutes or longer. Accelerometer data for the entire meeting period was considered valid if wearing time was equal to or greater than 30 minutes.

#### Girl Survey

Questionnaires assessed: 1) Fruit servings typically consumed. Several commonly consumed fruits were described, and children were given careful descriptions of how much of various fruits constituted a serving. One previously published item assessed typical servings of fruit per day: "On a typical day, how many servings of fruit do you eat [45]?" Responses were given on a five-point scale ranging from "none" to "4 servings or more"; 2) Vegetable servings typically consumed. This was assessed in a closely analogous manner to fruit, with one previously published item [45]. Fruits and vegetables were summed to create a single index of FV consumption. In a previous study, the measure significantly correlated with 3-day food recorded data and had good classification (63%) and specificity (63%) rates in child self-reports [46]. 3) Habitual PA. Physical activity was defined as "Any play, game, sport, or activity that gets you moving and breathing harder" and was discussed with numerous examples provided both by researchers and participants. Two items (α = .758) assessed days in the past week, and in a typical week (not counting physical education class) "On how many days were you physically active for a total of at least 60 minutes per day?" Responses were given on an eight-point scale ranging from "0 days" to "7 days." This measure has been previously validated in a diverse sample of adolescents [47]. 4) Sugar-sweetened beverage consumption was assessed with one item: "Over the past week, how often did you drink regular soda or sugar-sweetened beverages?" Responses were given on an eight-point scale, ranging from "never" to "every day" [48]. 5) Frequency of eating with television was assessed with three items (α = .658) from the Family Eating and Activity Questionnaire-Revised [48].

#### Parent Survey

Questionnaires assessed: 1) Family meals. Three items assessed FM frequency (breakfast, lunch, dinner, α = .665) from the Family Eating and Activity Questionnaire-Revised [48]. 2) Fruit servings and vegetable servings typically consumed by parent. Two items were identical to daughter's questionnaire. 3) Habitual PA of parent was assessed using four items from the Behavioral Risk Factor Surveillance System, assessing frequency and duration (in 10-minute bouts or longer) of moderate and vigorous PA [45]. 4) Frequency of eating while watching TV was assessed with three items (mother, father, child, α = .681) from the Family Eating and Activity Habits Questionnaire [48]. Example item is: "How often does the mother (female caregiver) eat while watching TV, reading, working?" Responses were given on a five-point scale, ranging from "Never" to "Always." 4) SSB consumption of parent was assessed with one item: "Over the past week, how often did you drink regular soda or sugar-sweetened drinks?" Responses were given on an eight-point scale ranging from "Never" to "Every Day." 5) Parent's height and weight were self-reported in feet, inches, and pounds. 6) Parenting style (potential moderating variable) was measured with the Parental Authority Questionnaire-Revised, containing 30 items, with three subscales indicating authoritative (democratic), permissive, and authoritarian (autocratic) parenting [49]. 7) The FACES II instrument's family cohesion subscale (16 items) was used to assess family cohesion (potential moderating variable) [50].

### Statistical Analysis

Intervention effects on individual outcome measures were analyzed consistent with other randomized controlled site-based interventions where the statistical design of the study is complicated by the lack of independence of data [38]. One girl's data are associated with other girls' data within troop sites (i.e., intra-class correlation). To adjust for the clustered data structure, a mixed-model design structure must be used, or the probability values will be incorrect. A full discussion of mixed-model analysis in group randomized trials with Girl Scout troops has been published elsewhere [22].

SAS 9.1 statistical software package (Cary, NC) was used for mixed-model analyses. SPSS 15.0 (Chicago, IL) was used to compute descriptive statistics and univariate analyses. To assess individual-level intervention effects, general linear model (PROC MIXED) analyses were run on difference scores (time 2 minus time 1), with girls nested within troop as random effect (to address clustering of girls within troops) and weight status (overweight or not), authoritarian parenting level (median split), socio-economic status (free/reduced or not) and race/ethnicity as fixed effects. To assess differences in objectively monitored physical activity by condition, general linear model (PROC MIXED) analyses were run on MVPA, with girls nested within troop as random effect, and weight status, socio-economic status, and race/ethnicity as fixed effects. To assess outcomes for troop meeting environments and percentages, descriptive statistics and chi-square analyses were used.

## Results

### Descriptive Information

Table 1 displays sample descriptive data by intervention and control conditions at time 1. There were no significant differences by condition for demographic variables.

### Process measures

#### Leader Self Ratings of Intervention Implementation

Three troop leader self-rating averages over the eight modules ranged from 1.52 to 1.86 (zero = no implementation to 2.0 = full implementation). Troops differed (F_2, 18 _= 21.5, p < .001) in overall implementation with averages of 1.43, 1.86, and 1.84 (mean = 1.71).

#### Troop Environmental Observations

Based on 2,328 minutes of direct observation data from 28 meetings, control troops spent about two thirds of meeting time devoted to Girl Scouting activities (67.3%). The remainder of control troop meeting time was spent on management (16%), snack (9.8%), active games (2.1%) and other content (4.9%). Based on 1,952 minutes of direct observation data from 21 meetings, intervention troops spent approximately 42.4% of meeting time devoted to Girl Scouting activities. The remainder of control troop meeting time was spent on active games (20.7%) management (17%), snack (18.3%), and other content (1.5%). Table 3 displays data for time spent in active games (physically active content) by study condition.

**Table 3:** Girl Scout troop meeting time in physically active content (4,280 minutes total observed time)

	Total minutes active content	Total observed minutes	Percent of minutes in active content	Mean minutes active content per meeting
**Intervention Troops**				
INT-1	99	824	12.0	14.1
INT-2	131	562	23.3	18.7
INT-3	175	566	30.9	25.0
mean	135	650.7	20.7*	19.3
				
**Control Troops**				
CON-1	0	394	0	0
CON-2	8	585	1.4	1.1
CON-3	30	742	4.0	4.3
CON-4	10	607	1.6	1.4
mean	12	582	2.1*	1.7

Note: INT = intervention troop; CON = control troop; *Significant difference by condition, x^2 ^= 210.8, p < .001

#### Snack

Table 4 displays raw frequencies and actual food exposures respectively by study condition. Food exposures were defined as the number of girls observed tasting/eating individual food products, regardless of amount eaten. Intervention troops provided a snack at 100% of meetings- consistent with the intervention policy, and control troops offered a snack at 71% of meetings. Intervention troops had greater opportunities for consumption of FV and drinking water. Control troops offered candy, cakes and cookies, and SSBs more often than intervention troops offered these foods.

**Table 4:** Raw frequency count of observed food accessibility and actual food exposures^ǂ ^in troop meeting snacks by condition (41 snack observations from 49 troop meetings)

	INT food accessibility	CON food accessibility	INT food exposures	CON food exposures
**Fruits and Vegetables**				
All fruits (with juices)	53	17	359	68
Fruit juices	14	9	94	39
All veget. (with juices)	33	6	225	30
Vegetable juices	2	0	11	0
				
**Drinks**				
Drinking water	12	0	89	0
Sugar-sweetened beverages	2	9	2	48
Other drinks	2	3	19	13
				
**Other food items**				
Salty Snacks	6	8	47	51
Dairy products (with milk)	13	7	93	51
Candy	1	13	6	72
Cakes and cookies	2	23	10	182
Breads	2	7	18	42
Meat, nuts, legumes	6	10	48	60
Condiments	4	0	30	0

Note: INT = intervention troop; CON = control troop; Intervention troops offered snacks at all 21 meetings, control troops offered snacks at 20 out of 28 meetings.

^ǂ^accumulated number of girls tasting/eating a food during troop meetings, over 41 troop observations

#### Meeting Environment and Leader Behavior

Table 5 illustrates the comparison between intervention troops and control troops in the meeting environment and leader behavior variables. Intervention meetings showed significantly more: Structured time (X^2 ^= 5.44, p = .020), physical activity knowledge content (X^2 ^= 6.38, p = .012), healthful eating knowledge content (X^2 ^= 13.64, p < .001), physical activity promotion (X^2 ^= 23.46, p < .001), and healthful eating promotion (X^2 ^= 18.14, p < .001). Control troop meetings were more likely to have no promotion of physical activity or healthful eating (X^2 ^= 1168.70, p < .001), and more likely to have leaders discouraging physical activity (X^2 ^= 4.64, p = .031) and healthful eating (X^2 ^= 5.88, p = .015). Finally, there was an insignificant trend for higher levels of family connection content (X^2 ^= 3.09, p = .079) in intervention troops.

**Table 5:** Troop environment and troop leader behavior by condition

	Intervention Troops	Control Troops	**X**^**2**^	Significance
Meeting time was structured (%)	97.4	90.3	5.44	P = .020
PA knowledge content (%)	6.0	0.3	6.38	P = .012
HE knowledge content (%)	11.7	0.4	13.64	P < .001
Family connection content (%)	3.1	0.0	3.09	P = .079
Any PA promotion (%)	16.6	1.5	23.46	P < .001
Any HE promotion (%)	18.9	0.4	18.14	P < .001
No PA or HE promotion (%)	64.5	99.1	1167.7	P < .001
Any PA discouragement (%)^a^	0.2	0.6	4.64	P = .031
Any HE discouragement (%)^a^	0	0.3	5.88	P = .015
No PA or HE discouragement (%)^a^	99.8	99.1	9.63	P = .002

Note: PA = Physical activity; HE = healthful eating; ^a^This X^2 ^calculated based on actual observed number, rather than percent

### Individual-level outcomes

#### Accelerometer-Measured Physical Activity

**Figure 2:**
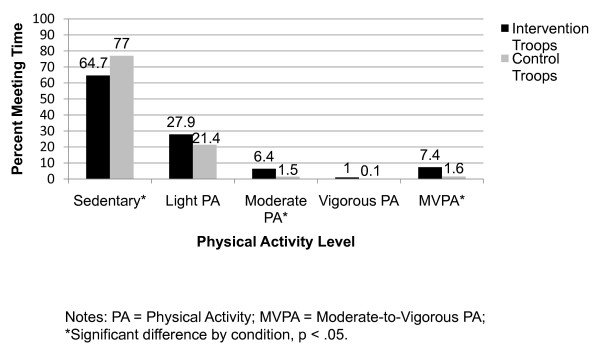
**Percent of troop meeting time spent at various intensity levels by condition, based on accelerometer data**.

Figure 2 displays accelerometer-measured physical activity levels of attending girls by condition. Girls in intervention troops accumulated significantly less sedentary (X^2 ^= 6.3, p = .011), significantly more moderate (X^2 ^= 8.2, p = .004), and moderate-to-vigorous physical activity (X^2 ^= 18.4, p < .001), than girls in control troops. Based on mixed-model analyses of accumulated minutes of MVPA, there were no significant differences by weight status (F_1, 400 _= 0.45, p = .50), by socio-economic status (F_1, 400 _= 1.86, p = .173), or by race/ethnicity (F_1, 400 _= 0.01, p = .924). There were no significant interactions between intervention and these categorical variables (F_1, 400 _= 0.01 to 0.21, p = .648 to .919).

Table 6 displays the adjusted mean scores at times 1 and 2, for intervention and control conditions. There were significant differences by condition at Time 1 for parent physical activity level and girl FV consumption. More parents in the intervention troops reported MVPA sufficient to meet recommended standards (X^2 ^= 8.87, p = .002). At time 1, girls in the intervention troops reported significantly higher intakes of FV (F_1, 73 _= 8.2, p = .005). No other variables differed significantly prior to randomization. All analyses of child outcomes are based on sample of 72 participants completing measures at times 1 and 2. Drop outs were low (n = 4, all from family relocation) and unrelated to troop assignment or the study.

**Table 6:** Individual outcomes at time 1 and time 2 by condition

Individual Outcome Variables	Time 1 INT Mean (SD)	Time 2 INT Mean (SD)	Time 1 CON Mean (SD)	Time 2 CON Mean (SD)
Girl's BMI	20.1 (4.4)	20.4 (4.5)	19.1 (2.9)	19.2 (3.0)
Girl's BMI percentile	65.2 (27.0)	64.8 (26.9)	64.5 (23.8)	62.2 (23.2)
Girl's BMI z-score	0.57 (0.94)	0.55 (0.94)	0.38 (0.75)	0.36 (0.74)
Parent BMI^a^	29.1 (6.4)	29.5 (6.9)	29.1 (6.7)	30.0 (7.5)
Family meals/week^a ^(scale 0-21)	11.2 (4.2)	10.9 (3.6)	11.4 (5.0)	12.1 (4.7)
Girl days/week of 60 min. MVPA^b ^(scale 0-7)	4.2 (1.8)	3.9 (1.7)	4.5 (1.9)	3.5 (1.8)
Percent of parents meeting MVPA standard^a^	46.7	42.0	33.3*	36.4
Girl FV servings/day^b ^(scale 0-8)	5.0 (2.0)	4.9 (1.7)	3.7 (1.9)*	3.7 (1.8)*
Parent FV Servings/day^a ^(scale 0-8)	3.9 (1.5)	4.4 (1.6)	3.7 (1.8)	4.4 (1.5)
Girl eating with TV scale^b ^(scale 0-4)	1.1 (0.7)	0.8 (0.7)	1.1 (0.8)	1.1 (0.7)
Parent eating with TV scale^a ^(scale 0-4)	1.8 (0.7)	1.7 (0.7)	2.0 (0.8)	2.1 (0.7)*
Girl days/week SSB consumption^b ^(scale 0-7)	3.1 (2.2)	2.3 (2.4)	2.6 (2.4)	2.2 (2.4)
Parent days/week SSB consumption^a ^(scale 0-7)	1.8 (2.2)	2.0 (2.6)	2.7 (2.7)	2.4 (2.8)

Note: Means adjusted for troop clustering, weight status, authoritarian parenting, socioeconomic status and race/ethnicity; INT = intervention; CON = control; BMI = body mass index; MVPA = moderate-to-vigorous physical activity; FV = fruits and vegetables;

TV = television; SSB = sugar-sweetened beverages; ^a^From Parent Report; ^b^From Child Report; *Significant difference by condition within same time point; For all variables, there were no significant differences in change from time 1 to time 2 by condition, p > .05.

#### Body Mass Index

The intra-class correlation coefficient assessing the troop-level variance associated with our primary outcome variable was low (ICC = .025). Mixed-model analysis on difference scores (T2 minus T1) revealed there were no significant main intervention effects for girl BMI z-scores (F_1, 5 _= 0.42, p = .544); or Parent BMI (F_1, 5 _= 1.58, p = .264). There was a significant main effect of socio-economic status on parent BMI (F_1, 35 _= 6.74 p = .014). Lower socio-economic status parents increased more than three BMI units from time 1 to time 2.

#### Influences on Body Mass Index, Girl

Table 6 displays the adjusted mean scores at times 1 and 2, for intervention and control conditions. Mixed-model analysis on difference scores revealed no significant main intervention effects for: Girl FV servings (F_1, 5 _= 1.54, p = .269); Girl MVPA (F_1, 5 _= 0.09, p = .779); Girl SSB consumption (F_1, 5 _= 0.41, p = .549); or Girl eating with TV, (F_1, 5 _= 0.63, p = .463). The analysis did reveal a significant main effect of socio-economic status on girl PA (F_1, 50 _= 8.18, p = .006). Lower socio-economic status girls decreased in PA from time 1 to time 2 across conditions.

#### Influences on Body Mass Index, Parent

Table 6 displays adjusted mean scores at times 1 and 2, for intervention and control conditions. Mixed-model analysis on difference scores revealed no significant main intervention effects for: Parent FV consumption (F_1, 5 _= 1.94, p = .223); Parent eating with TV (F_1, 5 _= 1.55, p = .269); Parent MVPA (F_1, 5 _= 0.87, p = .393); and Parent SSB consumption. This analysis revealed a significant main effect of socio-economic status on parent FV consumption (F_1, 5 _= 5.51, p = .023). Lower socio-economic status parents increased FV consumption by two servings from time 1 to time 2 across conditions. There was a significant main effect of authoritarian parenting style on parent PA (F_1, 46 _= 7.47, p = .009). Parents lower in authoritarian parenting level reported higher PA at time 2, compared to time 1. Aside from the lack of main intervention effects, there were significant interactions between socio-economic status and parent FV consumption (F_1, 47 _= 5.51, p = .023). Parents of lower socio-economic status in the control condition reported significant increases of FV consumption across times 1 and 2. There was a significant interaction between girl weight status and parent eating with TV (F_1, 50 _= 6.95, p = .011). Parents of overweight girls in the control condition increased eating with TV across the two time periods. Finally, there was a significant interaction between attendance level and intervention on parent PA (F_1, 50 _= 5.07, p = .029). The parents of girls with irregular attendance increased significantly in PA across the two time points.

## Discussion

The results of this study confirmed that troops randomly assigned to the intervention condition provided more leader promotion and opportunities for PA and healthful eating than control condition troops. Evidence of preferential effects from the intervention on obesity and its behavioral influences was limited solely to objectively measured PA levels of girls while attending troop meetings. Intervention girls were less sedentary and accumulated greater levels of MVPA than control girls. This is an important result, as it demonstrates the feasibility of implementing enjoyable opportunities for MVPA in what otherwise could be rather inactive troop meetings. With regard to public health, more physically active troop meetings could help attending girls to reduce sedentary behavior, and perhaps over time be more likely to achieve health benefits associated with meeting PA guidelines.

A major finding of this effectiveness study was that the intervention components were implemented with good fidelity in real-world settings, and resulted in troop leader health promotion behaviors and environmental opportunities for PA and healthful eating in the troop meetings. However, our intervention resulted in no measured impact on the behavioral influences of obesity for individual girls or parents in settings beyond the troop meeting environment. Intervention troop meetings offered ample physically active content and FV snacks, while control condition meetings offered very little PA or healthful eating opportunities. It appears that troop leaders delivered the curriculum, promoted PA, FM, and FV, discouraged SSB and eating with TV, and instituted troop policies in accordance with their training for the study. However, we saw no evidence for the hypothesized changes to BMI z-scores, habitual PA, FM, FV consumption, eating with TV, or SSB consumption. Our study's statistical power appears to be adequate for the primary outcome, but low for PA and FV. However, in all cases, there were not any identified trends in the data to suggest potential non-significant effects in these variables.

The lack of detectable effects beyond the troop environment may mean that the program itself was not efficacious outside the meetings, that the troop leaders were ineffective, or that girls failed to attend to the core messages and active learning opportunities, among numerous possibilities. It is also quite possible that our intervention had some effects that were not measured in our study. For example, improved skills, attitudes, behavioral intentions, empowerment, self efficacy, proxy efficacy, and increased knowledge of health promotion behaviors are possible outcomes that were not assessed in this study. According to a mediating variable framework, interventions impact mediating variables, which then act on behaviors and other outcomes [51]. Tests of mediation were beyond the scope of the paper, but further work using mediation analysis could help to answer the question as to why the intervention did not achieve measured outcomes beyond troop meetings. The intervention may have failed to impact potential mediators of behavior change, or perhaps the impacted mediators failed to result in detectable behavior change. Future studies of this sort should assess additional mediating variables such as skills, attitudes, intentions, and especially self efficacy for behaviors that impact obesity.

More than a decade ago, Cullen and co-authors conducted a nutrition education intervention in Girl Scout troops of similar age to the present study's sample [11]. Cullen found significant increases in FV consumption among the intervention troop scouts, and suggested that troop positive norms and social support could be created by consistently serving FV at troop meetings, which may lead to increased consumption of FV in scouts. Although we did not assess troop meeting environmental norms, per se, it appears that our intervention policies created a troop positive snack norm of having FV that nearly all the girls ate each time. Also, the healthful eating promotion efforts of the troop leaders could constitute social support, but we did not assess whether other girls were supportive of FV consumption. We were unable to detect positive outcomes on habitual consumption levels in our intervention troop girls, but better measures combined with a greater focus on FV, may have shown more favorable results. Similar to our approach, Baranowski and colleagues also used a customized badge incentive with Boy Scouts, and were able to increase FV consumption in their intervention [9,10].

On the PA side, intervention troop leaders provided girls with opportunities to be physically active at troop meetings, and accelerometer-based data show that the girls took advantage of those opportunities by being less sedentary and getting more MVPA than control girls. However, our data suggest that this bi-monthly troop meeting PA had no impact on girls' self-reported habitual PA levels. Similarly, Ievers-Landis and colleagues implemented an intervention to increase weight bearing PA (and calcium intake) in Girl Scouts, with a goal of primary prevention of osteoporosis [22]. The results of their study showed no significant differences in PA among two intervention groups and a control group. Future studies of such interventions may benefit by better measures, such as use of accelerometer-based measures of habitual PA.

The present study held a number of limitations, including a relatively small sample studied over only a five-month period. Our study's size and scope were limited based on constraints in funding. Despite the size, we believe the high participation rate, combined with extensive multiple observations, all bode well for the external validity of the findings within our geographic region. We relied on several self-report measures for both parents and children, and some measures may have limited reliability and validity. Similar studies in the future could be strongly improved with better measures of behavioral influences on obesity. In contrast to these limitations, our direct observation of troop environments and rigorous objective measurement of PA in troop meetings and child weight status provide major strengths for this study.

## Conclusions

In conclusion, the Healthier Troops in a SNAP intervention was implemented with good fidelity, and resulted in greater health promoting opportunities, MVPA, and healthful eating in troop meetings, but no discernable impact on the behavioral influences of obesity in children and parents outside of troop meetings. Implementing a health-promotion curriculum and supporting policies to provide more healthful environments in Girl Scouts troop meetings appears feasible on a broader scale. Fully powered intervention studies with additional measures of mediating variables will extend the work presented here. Improved approaches or coordinated interventions across environments may be needed to extend health promotion beyond scout meeting settings, if lasting behavior change and obesity prevention remain targeted outcomes.

## Abbreviations

BMI: body mass index; CON: control troop; FV: fruits and vegetables; HE: healthful eating; INT: intervention troop; MVPA: moderate to vigorous physical activity; PA: physical activity; SSB: sugar-sweetened beverages; TV: television.

## Competing interests

The authors declare that they have no competing interests.

## Authors' contributions

RR and DD conceived of the study. RR designed the SNAP intervention, oversaw data collection and analysis, and wrote first draft of the manuscript. DD and TB contributed to data analysis and revised manuscript drafts. All authors read and approved the final manuscript.

## Pre-publication history

The pre-publication history for this paper can be accessed here:

http://www.biomedcentral.com/1471-2458/10/81/prepub
